# Transthyretin amyloid deposits in lumbar spinal stenosis and assessment of signs of systemic amyloidosis

**DOI:** 10.1111/joim.13222

**Published:** 2021-01-07

**Authors:** P. Eldhagen, S. Berg, L.H. Lund, P. Sörensson, O.B. Suhr, P. Westermark

**Affiliations:** ^1^ Department of Medicine Karolinska Institute Karolinska University Hospital Solna Sweden; ^2^ Stockholm Spine Centre Löwenströmska Hospital Upplands Väsby Sweden; ^3^ Department of Public Health and Clinical Medicine Umeå University Umeå Sweden; ^4^ Department of Immunology, Genetics and Pathology Uppsala University Uppsala Sweden

**Keywords:** ATTR amyloidosis, cardiac amyloidosis, Lumbar spinal stenosis, magnetic resonance imaging, systemic amyloidosis

## Abstract

**Background:**

Wild‐type transthyretin (ATTRwt) amyloidosis is the most common systemic amyloidosis in Western countries and manifests mainly as progressive restrictive cardiomyopathy.

**Objective:**

To study the prevalence of ATTR deposits in ligament tissue in patients undergoing surgery for lumbar spinal stenosis and to assess whether these deposits are associated with cardiac amyloidosis.

**Materials and methods:**

A total of 250 patients, aged 50–89 (57% women), none with known cardiovascular disease, were included. Ligaments were investigated microscopically for amyloid. ATTR type was determined by immunohistochemistry and fibril type by Western blot. The amount of amyloid was graded 0‐4. All patients with grade 3‐4 ATTR deposits were offered cardiac investigation including ECG, cardiac ultrasound, plasma NT‐proBNP and cardiac magnetic resonance (CMR), including modern tissue characterization.

**Results:**

Amyloid was identified in 221 of the samples (88.4%). ATTR appeared in 93 samples (37%) of whom 42 (17 women and 25 men) were graded 3‐4; all had fibril type A (mixture of full‐length TTR and fragmented TTR). Twenty‐nine of 42 patients with grade 3‐4 ATTR deposits accepted cardiovascular investigations; none of them had definite signs of cardiac amyloidosis, but five men had a history of carpal tunnel syndrome.

**Conclusions:**

The prevalence of ATTR deposits in ligamentum flavum in patients with lumbar spinal stenosis was high but not associated with manifest ATTR cardiac amyloidosis. However, the findings of fibril type A, the prevalence of previous carpal tunnel syndrome and ATTR amyloid in surrounding adipose and vascular tissue indicate that amyloid deposits in ligamentum flavum may be an early manifestation of systemic ATTR disease.

## Graphical abstract

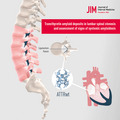

## Introduction

Systemic amyloidosis is a heterogeneous group of protein misfolding diseases where a protein is expressed in one or of few organs, secreted into blood plasma and transported to deposition sites where it aggregates into typical β‐sheet fibrils [1]. Transthyretin (TTR), a homotetrameric plasma protein synthesized mainly by the liver, gives rise to two types of systemic amyloidosis. One, which is hereditary, depends on missense mutation in the TTR gene, and more than 130 mutations are known. Most of them are associated with amyloidosis [2]. In the other, wild‐type (wt) ATTR amyloidosis (earlier designated senile systemic amyloidosis (1)), there is no TTR mutation [3, 4]. For the TTR to aggregate into fibrils, dissociation into monomers is necessary [5], and destabilization of the tetramer can occur either by mutations or by yet undefined mechanisms related to ageing. Whilst hereditary ATTR amyloidosis generally is a rare disease, the wild type is prevalent [6, 7, 8] and probably strongly underdiagnosed [9, 10].

Systemic ATTR amyloidosis can either consist of full‐length TTR molecules (type B) or a mixture of full‐length molecules and C‐terminal TTR fragments starting at around position 50 (type A) [11]. Only type A is associated with progressive restrictive cardiomyopathy [12]. Systemically appearing ATTRwt deposits are always of type A.

Lumbar spinal stenosis (LSS) is a clinical diagnosis where the spinal canal narrowing leads to compression of nerves leaving the cauda equina [13]. There are several causes, for example arthrosis and spondylolisthesis, but the underlying pathogenesis is unknown. The thickening of connective tissue, including ligaments, particularly ligamentum flavum, is common. LSS is a common condition, occurring in both sexes and with increasing prevalence with age. It creates severe disability for the patient and economic loss for society. Treatment can be conservative, but surgical decompression is often indicated. Recurrence of symptoms with the need for reoperation is frequent [14].

The finding of amyloid in the transverse ligament of the wrist in association with carpal tunnel syndrome is well known. It was first linked to a haemodialysis‐associated Abeta2‐microglobulin form of systemic amyloidosis [15]. More recently, it has been pointed out that carpal tunnel syndrome commonly precedes ATTR amyloidosis, both hereditary and wild type, sometimes by many years [16, 17, 18, 19, 20]. Amyloid deposits are common in the transverse ligament and in different joint tissues including ligaments and cartilage [21, 22] and in connective tissue in the vertebral column [23]. The age‐dependent occurrence of amyloid in the ligamentum flavum has been described [24, 25]. The amyloid nature has been studied only recently, and immunohistochemistry has identified TTR in some reports [26], but apolipoprotein A‐I has also been found [27].

There are scattered reports on patients with amyloid in ligamentum flavum in specimens obtained at the surgery for LSS [24]. In a pilot study of resected material from 26 patients undergoing surgery for LSS, 10 contained amyloid [28]. Immunohistochemistry indicated the presence of TTR in the deposited material in 5 patients. The present study was conducted (1) in a large cohort of patients undergoing surgery for LSS, to assess the prevalence of ATTR deposits in ligamentum flavum; and (2) amongst patients with ATTR deposits to evaluate the prevalence of cardiac involvement and/or carpal tunnel syndrome.

## Materials and methods

### Patients

Consecutive patients, 50 years old and older of both sexes accepted for surgery for symptomatic lumbar spinal stenosis, were between May 2016 and October 2018 invited to participate in the study. Only patients with American Society of Anesthesiologists (ASA) class 1 and class 2 were accepted for surgery at the participating surgical clinic. Patients with ASA class 3 or higher including moderate‐to‐severe comorbidities such as previous pacemaker, known heart failure, renal impairment, ischaemic heart disease or cardiomyopathy were not accepted; hence, patients in this study had no previous medical history of significant cardiovascular disease, even if this was not an exclusion criterion for the study.

The LSS diagnosis was confirmed by magnetic resonance imaging (MRI) in which narrowing of the spinal canal at one or several levels was required for diagnosis. The study was approved by the Ethical Committee at Uppsala University Hospital and conducted according to the Declaration of Helsinki. Written consent was obtained from all patients.

### Tissue material

During surgery, decompression of the narrowing was performed by tissue removal. Removed fragments of ligamentum flavum and other ligaments were put in 0.15 M NaCl and transferred to the amyloid laboratory. Small pieces (usually 3 about 10 × 5 × 2 mm) of ligament tissues were taken at random, fixed in 4% buffered neutral formaldehyde solution and embedded in paraffin, and the rest of the material was stored frozen at −20°C.

### Amyloid and amyloid proteins

**Fig. 1:**
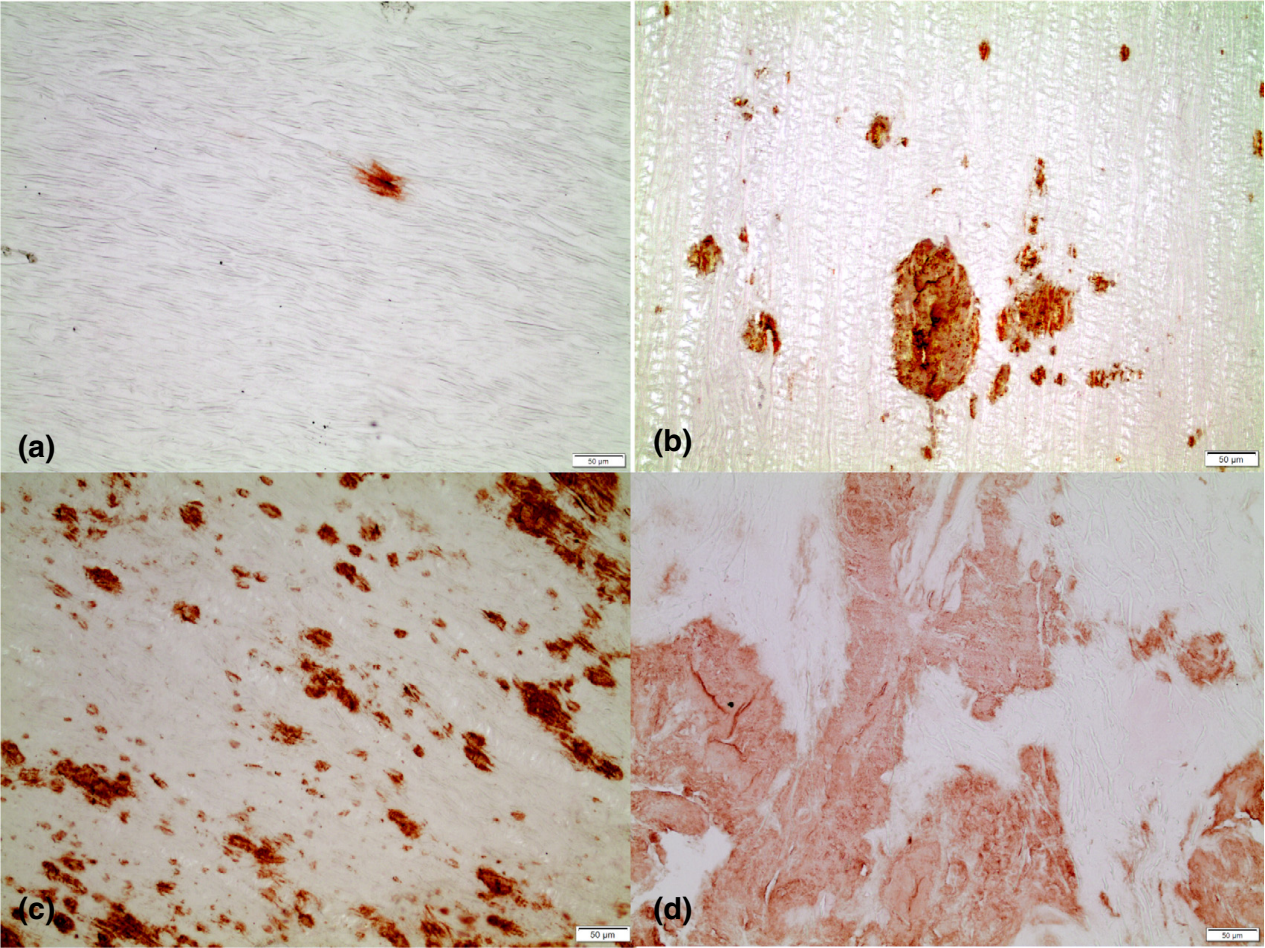
Visualization of the grading system of transthyretin amyloid deposits: (a), grade 1; (b), grade 2; (c), grade 3; and (d), grade 4. Amyloid deposits labelled with monoclonal antibody 7×. Bar = 50 µm.

One section of ligament tissue from each patient was stained with Congo Red and examined in a polarization microscope for amyloid. Other sections were first immunolabelled with the mouse monoclonal amyloid antibody (MAB) 7X, developed against C‐terminal ATTR fragments purified from heart amyloid. This MAB recognizes both full‐length TTR and C‐terminal TTR fragments and labels ATTR deposits specifically [29]. The immunolabelled sections were then stained with Congo Red, which made it possible to identify both ATTR amyloid and amyloid deposits composed of other proteins in the same sections. The amount of ATTR amyloid was scored according to a semiquantitative scale in which 1 = few small scattered deposits; 2 = scattered but not confluent deposits or a single large deposit in one fragment; 3 = widely spread, moderately large deposits in all ligament tissue or large, confluent deposits in more than one fragment; and 4 = widely spread, large confluent deposits (Fig. 1).

The presence of non‐ATTR‐labelled deposits was recorded but not graded. ATTR deposits in blood vessel walls or in fat tissue, sometimes present in surrounding tissue, were also recorded.

Material from ligament tissue from all patients with grade 3 and grade 4 ATTR deposits was subjected to sodium dodecyl sulphate polyacrylamide gel electrophoresis (SDS‐PAGE) and Western blot analysis [30]. New small ligament pieces were thawed, washed with distilled water followed by acetone, dried and boiled directly in sample buffer. After SDS‐PAGE and transfer to a nitrocellulose membrane, ATTR was identified by a polyclonal rabbit antiserum 1898 raised against recombinant human TTR50‐127, which recognizes both full‐length TTR and C‐terminal TTR fragments in Western blot [31]. The reaction was visualized as described [32].

### Cardiac investigations of patients with 3‐4 ATTR deposits

All patients with 3‐4 ATTR deposits were contacted by phone and offered a visit at the Karolinska University Hospital, including cardiovascular investigation with ECG, medical history, physical examination, NT‐proBNP, echocardiography with strain analysis and cardiovascular MRI (CMRI; Siemens 1 5T) with gadolinium contrast. CMRI images were used for assessing left ventricular function, volumes and mass, late gadolinium enhancement (LGE) for focal fibrosis detection, midventricular septal analyses of native T1 (oedema/amyloid deposits) and extracellular volumes (ECV) for diffuse fibrosis [33, 34].

As part of the preoperative procedure at the surgical unit, ECG was taken in patients on a clinical basis, and all available ECGs in this cohort were collected and analysed for PR interval, QRS duration and rhythm disturbances, for example atrial fibrillation.

### Statistical methods

Statistical analyses were performed with GraphPad Prism 7.02C program. Continuous data are reported as mean ± SD and categorical data as number and median. Multiple comparisons of continuous data were performed by one‐way ANOVA with Tukey’s post hoc test. Categorical data were analysed by Fisher’s exact test. A *P*‐value < 0.05 was considered significant.

## Results

### Patients

**Fig. 2:**
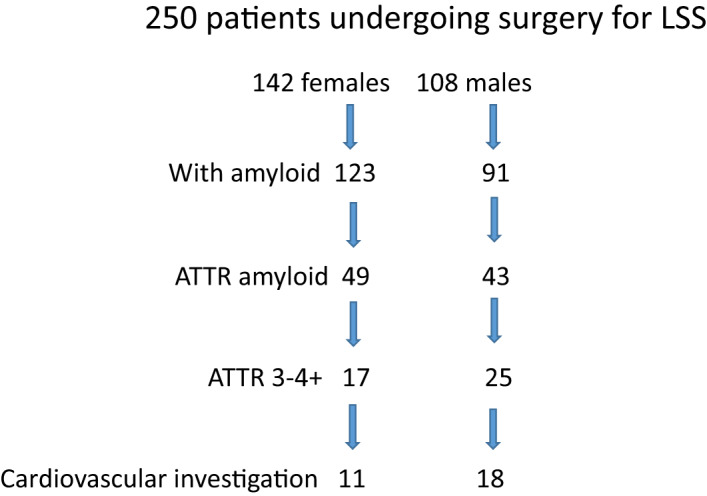
Flow chart.

A total of 250 patients were included in the study; see flow chart, Fig. 2. The mean age at surgery was 67.8 ± 8.0 (range: 51–89 years). Women predominated (*n* = 142; 56.6%). There was no significant difference in age between women and men (mean: 68.3 ± 8.3 and 67.2 ± 7.1, *P* > 0.2). The distribution in different age categories is displayed in Table 1.

**Table 1:** Transthyretin amyloid identified in 250 patients undergoing surgery for lumbar spinal stenosis

	Amyloid degree									
	Females					Males				
Age (years)	0	1	2	3	4	0	1	2	3	4
50–59	23	2	0	0	0	16	1	1	2	0
60–69	44	6	4	1	0	29	3	3	5	0
70–79	22	8	9	10	3	19	7	4	13	3
80–89	4	0	3	1	2	0	0	0	2	0

### Amyloid prevalence

Amyloid deposits were found in 221 of the 250 samples (88.4%). Patients without any amyloid in the ligamentum tissues were younger than those with (61.1 ± 1.1 vs. 68.2 ± 0.6; *P* < 0.02), but there was no difference in sex distribution.

**Fig. 3:**
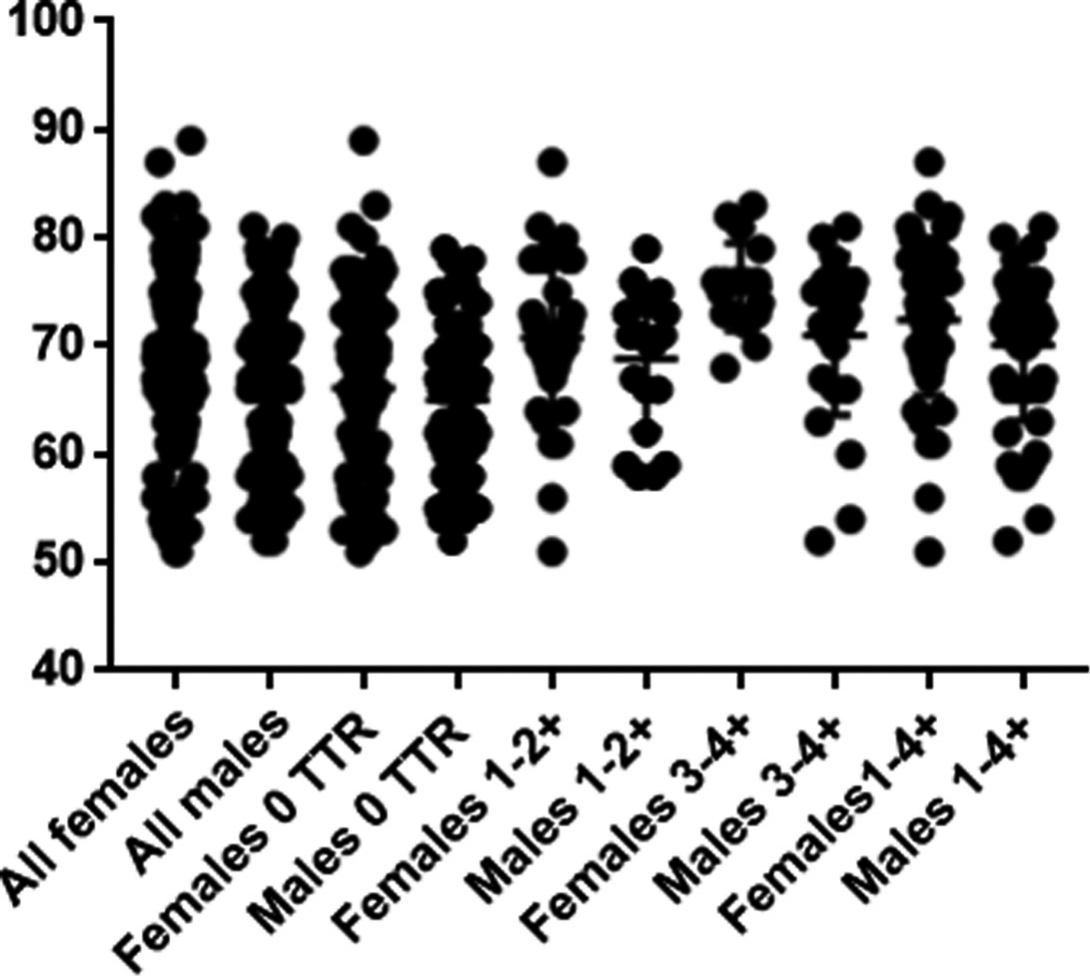
All patients with and without transthyretin amyloid deposits in lumbar ligament tissue. The *y*‐axis represents age.

Occurrence and scoring of ATTR content in the different groups are shown in Fig. 3. ATTR amyloid deposits of any score were identified in 93 patients (37.2%), and there was no significant difference between women and men. There were significantly more men than women with grade 3‐4 ATTR deposits (*P* < 0.03). Furthermore, males with grade 3‐4 ATTR amyloidosis were significantly younger than females with a similar degree of deposition (71.1 ± 1.4 and 75.5 ± 1.0 year, respectively, *P* < 0.03). There were no statistical differences in age between women and men in the groups with 1‐2 ATTR or no deposits. ATTR amyloidosis was significantly more prevalent in the 70‐ to 79‐year age group than in the younger age groups in both women (*P* < 0.0001) and men (*P* < 0.002).

### Amyloid appearance

**Fig. 4:**
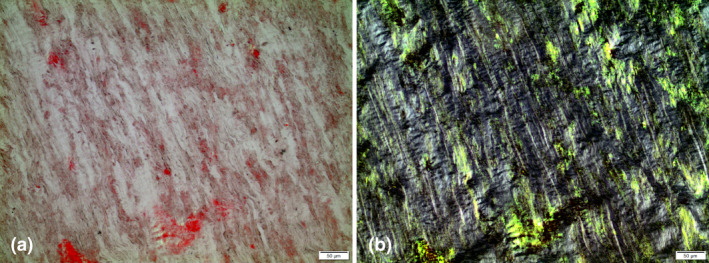
Ligament transthyretin amyloid stained with Congo Red, (a) without crossed polars and (b) between crossed polars. The typical birefringence is seen in (b). Bar = 50 µm.

**Fig. 5:**
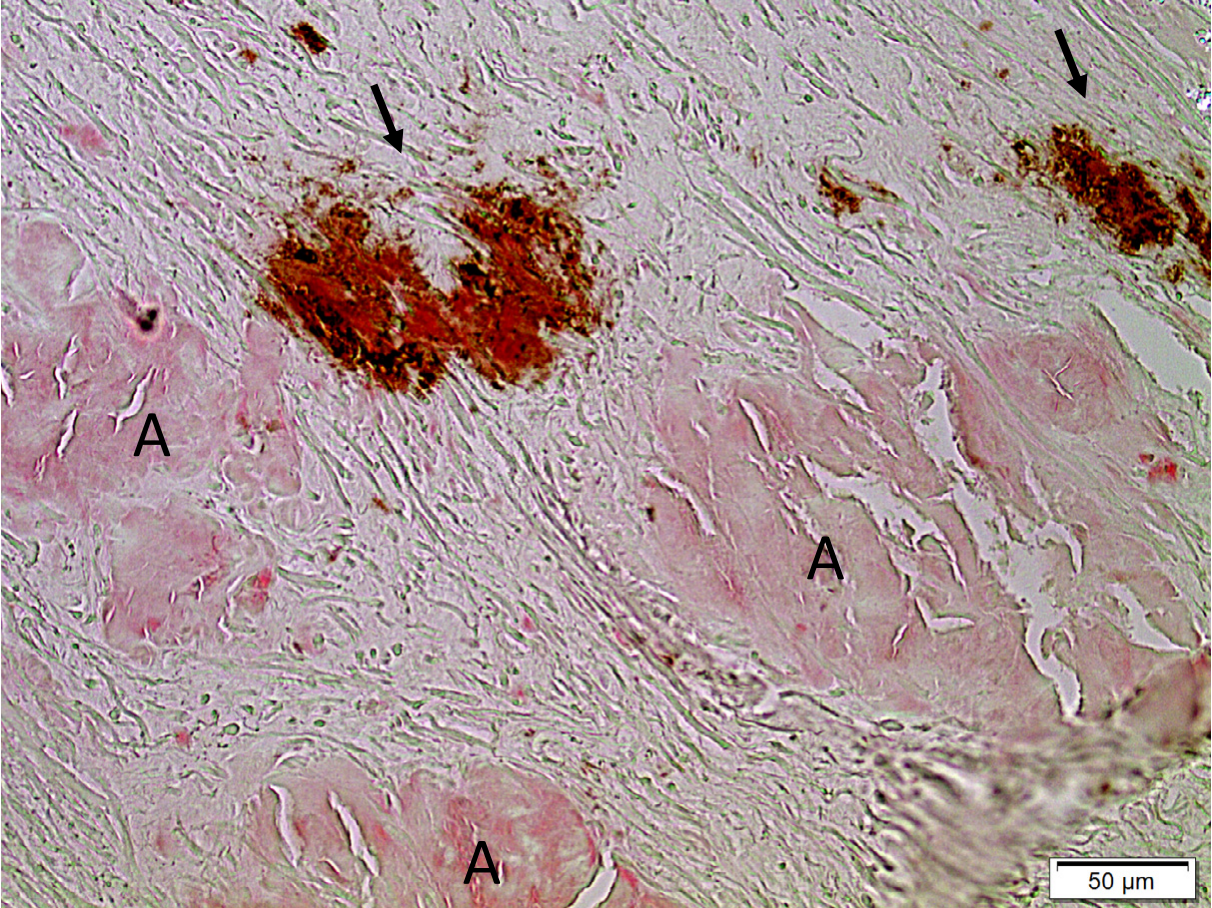
Ligament tissue double‐stained for amyloid with Congo red and for immunohistochemical detection of transthyretin amyloid (ATTR) by monoclonal antibody 7×. ATTR is stained brown (arrows), but in this case an additional type of amyloid of unknown composition is abundant (A). Bar = 50 µm.

Amyloid was generally easily identified by a strong affinity for Congo red and characteristic birefringence of varying colours (Fig. 4), but there were some exceptions. Very weakly Congo red‐stained amyloid appeared in a few cases, sometimes in the form of thin fibres. Amyloid deposits varied in morphology. Multiple distinct areas were most common, and these were sharply demarcated against surrounding ligament structures (Fig. 5). Their size varied from very small to large masses.

ATTR positivity was strong, and deposits in most cases with extensive amyloidosis were of ATTR nature. Large, coalescent amyloid was always labelled with ATTR antibodies. However, in a majority of materials with ATTR amyloid deposits, there were also Congo red‐positive and birefringent areas that did not label with the ATTR antibodies. These other deposits were morphologically varying but sometimes indistinguishable in shape from ATTR amyloid (Fig. 5). Very often, such material was in direct contact with ATTR amyloid, and a mixture of ATTR‐positive and ATTR‐negative areas was a common finding.

### Amyloid in blood vessels and adipose tissue

**Fig. 6:**
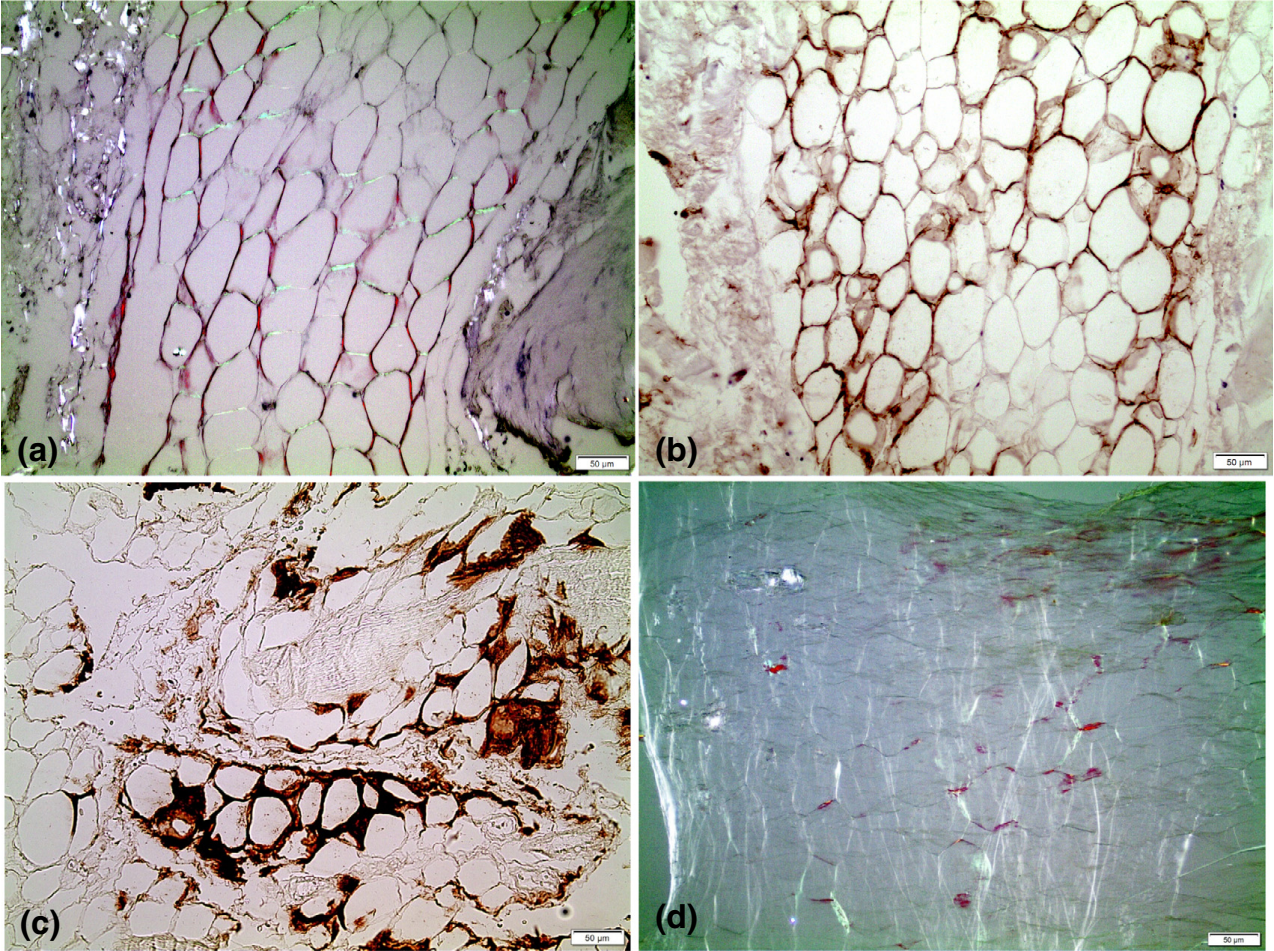
Adipose tissue in association with ligament tissue, in (a and d) stained with Congo red and examined with partially crossed polars, a method that enhances the red colour. (a) is from a section of ligament tissue, whilst (d) is from fat tissue particle. In panels (b and c), amyloid in adipose tissue from two different patients is visualized immunohistochemically with monoclonal antibody 7×. Brown colour demonstrates the transthyretin amyloid. Bar = 50 µm.

The investigation was only planned for study of amyloid in ligament tissue, and therefore, only minor other tissue components appeared in the sections. In 10 samples from patients with grade 3‐4 ATTR deposits, adipose tissue was recognized, and in 4 of them (2 females, 76 and 75 years, and 2 males, 76 and 80 years), ATTR amyloid appeared with a distribution similar to what is seen in subcutaneous or pericardial fat tissue in patients diagnosed with systemic ATTR amyloidosis (Fig. 6a, b and c). Based on this finding, efforts were undertaken to identify adipose tissue in residual material stored frozen in distilled water. After thawing, small fat particles were found free‐floating in 14 of the 42 ATTR 3‐4 patient residual materials. These particles were prepared, stained and analysed between crossed polarizers in the same way as subcutaneous adipose tissue biopsies [35]. Amyloid was present in 6 (43%) of these materials (Fig. 6d).

A few blood vessels were identified in ligament from 3 patients with 3‐4 ATTR deposits (2 males, 76 and 80 y, and one female, 81 y), and in all these vessel walls, ATTR amyloid was seen.

### Characterization of ATTR in ligamentum flavum

**Fig. 7:**
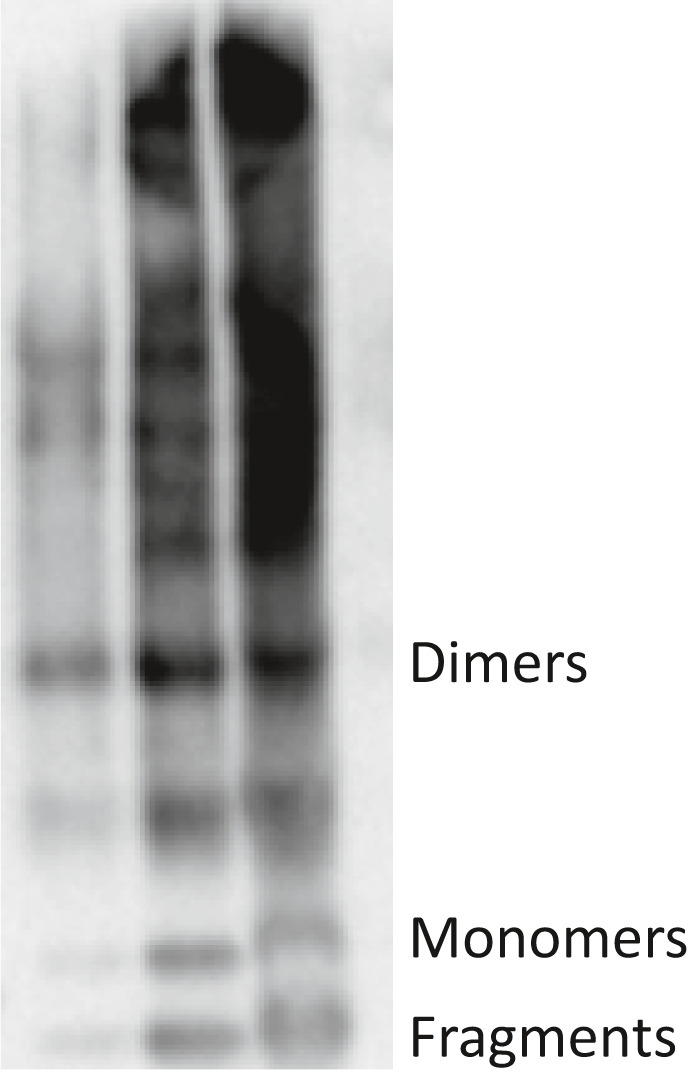
Western blot analysis of extract of three degree 3 + ATTR ligament materials with the aid of in‐house rabbit antiserum 1898. Typical transthyretin amyloid bands, including fragments, are denoted. The heavy smear at higher molecular weight species is due to aggregates.

Extracts of small pieces of ligamentum flavum from all 42 patients with grade 3‐4 ATTR amyloid were analysed by Western blot using an antiserum (1898) that recognizes both full‐length and C‐terminal ATTR fragments [36, 37]. All materials from 42 patients had the typical pattern A appearance in the blots with distinct fragment bands in addition to full‐length ATTR bands (Fig. 7), indistinguishable from what is seen in ATTR extracted from systemic ATTR type A amyloidosis.

### Cardiovascular findings

Out of the 250 patients included, an ECG was analysed in 149 patients. The ECG was taken either preoperatively or later at the cardiology visit. Some patients had multiple ECGs, in which case only the first recorded ECG was analysed. PR interval > 220 ms, QRS duration > 120 ms or the presence of atrial fibrillation was considered an abnormal ECG. ECG was abnormal in 7/40 (17%) of patients with grade 3‐4 ATTR deposits compared to 10/109 (9%) of patients without or with lower grades of ATTR deposits (*P* = 0.24). ECG abnormalities were significantly more common in men than in women (*P* = 0.001), and amongst males, there was no significant difference in ECG findings depending on the grade of ATTR deposits (*P* = 1.0).

Twenty‐nine out of 42 (69%) of the patients with grade 3‐4 ATTR deposits accepted a visit with cardiovascular investigations, and characteristics are shown in Table 2. CMR was performed in 26 patients and echocardiography in all 29 patients, of which a complete analysis was possible in 24.

**Table 2:** Clinical findings in 29 patients with degree 3‐4 transthyretin amyloid deposits in lumbar ligament tissue

	Men *n* = 18	Women *n* = 11	Total *n* = 29
Age, y (range)	73 (63–82)	74 (69–88)	74 (63–88)
Surgery for carpal tunnel syndrome^a^	5 (28)	0	5 (17)
Glaucoma (%)	3 (17)	0	3 (10)
Polyneuropathy	2 (11)	0	2 (7)
Atrial fibrillation/flutter	1 (6)	0	1 (3)
Ischaemic heart disease	1 (6)	0	1 (3)
Aortic valve disease	1 (6)	0	1 (3)
Stroke	0	1 (9)	1 (3)
Symptoms from lumbar spinal stenosis (median y)	3	5	3
NYHA^b^ class 1	12 (63)	4 (36)	16 (53)
NYHA class 2	7 (37)	6 (55)	13 (43)
NYHA class 3	0	1 (9)	1 (3)
Hypertension	10(56)	8 (73)	18(62)
Type 2 diabetes	5 (28)	0	5 (17)

^a^ Number of patients; percentage in parenthesis.

^b^ New York Heart Association.

On echocardiography, median septal thickness was 11 mm (range: 8–15). 11/24 (46%) patients had a septal thickness of at least 12 mm.

On CMR, left ventricular mass, volumes, systolic function, septum midventricular analyses of native T1 and ECV were normal in all patients. Small areas of focal late gadolinium enhancement were noted in 6/18 men (33%) but in none of the 8 women (*P* = 0.098) (Table 3).

**Table 3:** Findings at cardiac MRI in 26 patients with grade 3‐4 ATTR amyloid deposits

Age, years	74 (72–76)
Height, cm	174 (170–184)
Weight, kg	79 (75–89)
BSA, m^2^	2.0 (1.8–2.1)
LVEDV, mL	146 (133–167)
LVEDVi, mL m^−2^	77 (68–86)
LVESV, mL	63 (53–72)
LVESVi, mL m^−2^	32 (26–38)
LVSV, mL	83 (77–95)
LVSVi, mL m^−2^	43 (41–46)
LVEF, %	58 (55–60)
LVM, mL	127 (108–147)
LVMi, mL m^−2^	62 (58–74)
ECV, %	26 (24–28)
T1	977 (960–1008)
Focal LGE, *n* (%)	6 (23)

Body and CMR measures presented as median (inter‐quartile range). CMR, cardiac magnetic resonance, LVEDV(i), left ventricular end‐diastolic volume (indexed to BSA); LVESV (i), left ventricular end‐systolic volume(indexed to BSA); LVSV (i), left ventricular stroke volume(indexed to BSA); LVEF, left ventricular ejection fraction ; LVM(i), left ventricular mass(indexed to BSA); ECV, extra cellular volume; LGE, late gadolinium enhancement.

Nt‐proBNP was measured in 28 patients. A value >125 ng L^−1^ was measured in 13/28 patients (median: 120, range: 12–545).

Five out of the 18 men (28%; 76, 66, 74, 67 and 80 years old) had a history of previous surgery for carpal tunnel syndrome of which 4/5 were bilateral. None of the 11 women had such a history. However, the difference is not statistically significant.

## Discussion

This study is, to our knowledge, the most extensive study of amyloid deposits associated with lumbar spinal stenosis. This unique study showed a very high prevalence (37%) of ATTR amyloid deposits in vertebral ligament tissue in patients aged over 50 undergoing lumbar spinal stenosis surgery. In 42 patients (16.8%), the amount of amyloid was of grade 3‐4 on a 4‐grade visual scale.

It could be argued that we should have performed a cardiologic investigation in all 250 patients since, in ATTR amyloidosis, there is no definite relationship between amyloid amounts in different organs. We reasoned that it is more likely to find cardiac involvement in patients with pronounced deposits in ligament tissue and investigated only these patients further in this study. We got some support for our assumption when the study of ligament material was finished: amyloid in blood vessels and around fat cells was seen in sectioned material of six grade 3‐4 patients but only in one of grade 2 and none in grade 1 or grade 0.

A central question is whether ligament ATTR deposits are localized to this tissue or part of systemic disease. Since patients with a significant history of cardiac disease were not accepted for surgery at the surgical unit, no patients with known ATTRwt amyloid cardiomyopathy were to be included in the study.

The observed prevalence of septal thickness 12 mm or more on echocardiography that may be a sign of cardiac amyloidosis was not consistent with CMR findings that all showed normal ventricular mass and no hypertrophy. CMR has been shown to be a more accurate method to assess left ventricular mass [38]. Hence, the finding on echocardiography was considered not indicative of cardiac amyloid.

In spite of no clear evidence of clinical cardiac amyloidosis in the 42 investigated patients, three different facts and findings indicate that ATTR deposits in the ligament tissue in LSS may be part of systemic amyloidosis. First, fibril proteins of localized amyloid forms, for example Aβ in brain and IAPP in islets of Langerhans, are synthesized by cells close to deposition sites, whereas fibril proteins in systemic amyloidosis are synthesized far from the deposition site, to where they are transported by the blood [1]. There is no known expression of TTR in or close to ligament components. Second, by examination of surrounding tissue in ligaments with degree 3‐4 deposits, ATTR was found around fat cells in a similar pattern to that of systemic ATTR disease, for example in subcutaneous or pericardiac tissue or blood vessels. Third, in 5 male but no female patients, CTS was reported, a diagnosis that is a common early sign of ATTRwt disease [17], a predominantly male disease. Therefore, it seems reasonable to suspect that a substantial group of our patients had developed a systemic disease, albeit in an early stage.

Ligament tissue may be the first place for ATTR fibrillogenesis by yet unknown mechanisms. From this place, amyloid later may disseminate throughout the body by seeding [39]. Seeding is an essential mechanism by which misfolded proteins spread and induce amyloid formation at other sites [40]. A mechano‐enzymatic mechanism has been proposed for ATTR amyloidogenesis in the heart [41] and is an attractive hypothesis for ligaments since the hypothesis involves a steady tissue movement. A recent cryo‐electron microscopic study of native type A ATTR fibrils indicates a more or less complete TTR unfolding during amyloidogenesis [42]. However, the study was based on fibrils from a patient with a V30M TTR mutation and no similar investigation of wild‐type ATTR fibrils has been published.

A great majority of patients with advanced cardiac ATTRwt amyloidosis are men, but there was no significant difference in prevalence between women and men in the grade 3‐4 categories in our material. However, women may develop severe deposits later since no female in the present study was under 72 year whilst there were 10 males, 53–71 year old. Again, it is notable that 5 men and no woman had a history of CTS.

Another critical question is whether ATTR amyloid has any clinical relevance in ligament tissue. In one study, the thickness of ligamentum flavum correlated with the amount of amyloid [43]. In the study on osteoarthritis by Rubin et al. [44], no examination of the joint tissue was performed, but it was hypothesized that amyloid deposition could be directly pathogenic. This hypothesis was supported by a mouse model of osteoarthritis, where it was shown that the overexpression of TTR worsened the progression of the disease [45]. Taken together, the three studies indicate that ATTRwt amyloidosis is not only a cardiac disease.

Amyloid deposits appeared in 221/250 (88%) of the specimens, but only in 93/250 (37%) a TTR nature was verified based on studies with two different ATTR antibodies. Consequently, at least one more kind of amyloid must be present as shown earlier (28, 43). In a majority of the ATTR‐positive materials in our study, additional types of amyloid were seen. The nature of the other deposits is unknown although apolipoprotein AI has been identified previously as a probable fibril component. However, with the use of immunohistochemistry, we were not able to identify this protein in the present study. If the different amyloids develop independently or if their pathogeneses are interrelated is unknown. Notable was the common finding of these non‐ATTR amyloid deposits at all ages.

In conclusion, 93 out of 250 patients (37%) with lumbar spinal stenosis had ATTR deposits in removed ligament tissue, and amongst these, 42/250 (16.8%) were graded 3‐4. Cardiovascular investigation in 29 of the patients with grades 3‐4 did not identify anyone with definite cardiac amyloidosis using modern CMR protocol with tissue characterization. It is still possible that ATTR deposits in ligament tissue are an early event in systemic disease and that some of the patients will develop clinically significant cardiac amyloidosis later. The incidence of cardiac amyloidosis after surgery for lumbar spinal stenosis needs further studies.

## Limitations

Technetium 99m labelled 3,3 diphospono‐1,2 propanodicarboxylic acid (DPD) scintigraphy, which is a specific investigation for cardiac ATTR amyloidosis, was not part of the study protocol. DPD scintigraphy may be relevant in the follow‐up of patients with localized deposits, such as in our study. Our study excluded patients with significant cardiac comorbidities, and only patients with ASA class 1 or class 2 were accepted for surgery. If we had included patients with concomitant heart disease, the results might have been different, but we could not have answered whether cardiac involvement was prevalent or incident after symptoms of LSS occurred.

We did not investigate patients with grade 1‐2 ATTR deposits in ligament further in this study. If the actual grade (0‐4) of ATTR amyloid deposits is of importance as a risk marker for systemic amyloidosis is unknown.

## Conflict of interest statement

Dr. Eldhagen reports grants and personal fees from Pfizer, personal fees from Alnylam, Orion Pharma and Sanofi, outside the submitted work. Dr. Lund reports personal fees from Merck, Sanofi, Bayer, Pharmacosmos, Abbott, Medscape and MyoKardia; grants and personal fees from Vifor‐Fresenius, AstraZeneca, Relypsa, Mundipharma, Boehringer Ingelheim and Novartis; and grants from Boston Scientific, outside the submitted work. Dr. Suhr reports nonfinancial support and other from Pfizer Pharmaceuticals, Prothena Pharmaceuticals, Alnylam Pharmaceuticals and Akcea Pharmaceuticals and other from Eidos Therapeutics, Inc, and Intellia Pharmaceuticals, outside the submitted work. Dr. Westermark reports personal fees and research grants from Pfizer and Alnylam. Dr. Berg and Dr. Sörensson have nothing to disclose.

## Author contribution

**Per Eldhagen:** Conceptualization (equal); Data curation (equal); Formal analysis (equal); Writing‐original draft (equal). **Svante Berg:** Conceptualization (equal); Data curation (equal); Investigation (equal); Resources (equal); Writing‐original draft (equal). **Lars H. Lund:** Formal analysis (equal); Resources (equal); Writing‐review & editing (equal). **Peder Sörensson:** Data curation (equal); Formal analysis (equal); Investigation (equal); Methodology (equal); Writing‐review & editing (equal). **Ole Suhr:** Conceptualization (equal); Writing‐original draft (equal); Writing‐review & editing (equal). **Per Westermark:** Conceptualization (equal); Funding acquisition (lead); Project administration (equal); Resources (equal); Writing‐original draft (equal); Writing‐review & editing (equal).
